# Stapling cartridge lavage cytology in limited resection for pulmonary malignant tumors: assessment of cytological status of the surgical margin

**DOI:** 10.1016/j.heliyon.2019.e01240

**Published:** 2019-02-15

**Authors:** Tomohiro Miyoshi, Junji Yoshida, Keiju Aokage, Kenta Tane, Genichiro Ishii, Masahiro Tsuboi

**Affiliations:** a Division of Thoracic Surgery, Department of Thoracic Oncology, National Cancer Center Hospital East, 6-5-1, Kashiwanoha, Kashiwa, Chiba, 277-8577, Japan; b Division of Pathology, Exploratory Oncology Research and Clinical Trial Center, National Cancer Center, 6-5-1, Kashiwanoha, Kashiwa, Chiba, 277-8577, Japan

**Keywords:** Oncology, Surgery

## Abstract

**Introduction:**

Sublobar resection in primary lung cancer and pulmonary metastatic tumor can result in recurrence at the surgical margin. Confirming the absence of tumor cells at the cut-end is important. We sought to evaluate the efficacy of intraoperative lavage cytology (ILC) of autostapling cartridges in preventing local failure.

**Materials and methods:**

An intraoperative cytology examination was performed in 262 consecutive patients undergoing wedge or segmental resection for 311 lesions, including primary lung cancers and pulmonary metastatic tumors, between April 2004 and April 2010. The data of patients with positive cytology results and those who developed local failure were retrospectively reviewed.

**Results:**

A total of 139 primary lung cancers and 172 pulmonary metastatic tumors (primary site: 120 colorectal and 52 others) were investigated. The results revealed 22 (7%) positive cytology results (11 primary and 11 metastatic). The resection margins of 19 of the 22 lesions with positive cytology were additionally resected. With a median follow-up period of 42 months, recurrence at the margin developed in 2 of the 19 lesions in which additional resection was performed (11%, 1 primary and 1 metastatic). Recurrence at the margin developed in 2 (67%, 1 primary and 1 metastatic) of the 3 lesions in which additional resection was abandoned. Among the 289 lesions showing negative cytology results, recurrence at the margin developed in 7 (2%, 6 primary and 1 metastatic).

**Conclusions:**

ILC of autostapling cartridges in sublobar resection for pulmonary malignant tumor may be useful for assessing the cytological status of the surgical margin.

## 1 Introduction

Limited surgical resection of the lung (i.e., less than lobectomy) has the advantage of better postoperative lung function due to preservation of the lung parenchyma [1, 2, 3]. For that reason, limited surgical resection is employed in patients with lung cancer and impaired pulmonary function, as well as in elderly patients unfit for lobectomy [4]. Simultaneous or metachronous multiple primary lung cancers are also indicated to undergo limited lung resection to preserve the lung parenchyma. Along with the recent increase in the incidence of peripheral small-sized lung adenocarcinoma, which has favorable prognosis, such early lung cancers have been aggressively managed with limited surgical resection [1, 3, 5, 6, 7, 8, 9, 10, 11].

Pulmonary metastasectomy for patients with colorectal cancers has been reported to be associated with a favorable prognosis [12]. Limited surgical resection is the preferred procedure of choice in metastasectomy for pulmonary metastatic tumor because of the high risk of new metastasis after pulmonary metastasectomy.

Although limited lung resection has the advantage of preserving lung parenchyma, an increased risk of local recurrence at the surgical margin has been observed [12, 13, 14, 15, 16, 17, 18, 19], even in patients whose macroscopic resection margin was sufficiently secured. To prevent local failure, several methods such as frozen section analysis or stamped cytologic examination were reported to assess residual tumor cells at the cut-end during surgery [20].

Frozen section analysis is a standard way to investigate the cytological status of the surgical margin. However, the furthermost lung parenchyma that should be evaluated must be removed with the stapler, and the measured length does not always represent the shortest one because measurements are performed on only one microscopic slide.

Higashiyama et al. showed that employing intraoperative lavage cytology (ILC) of autostapling cartridges used to cut lung parenchyma was effective in reducing recurrence at the resection margin [15, 16]. They speculated that ILC of autostapling cartridges was superior to conventional techniques because the entire area of the margins can be collectively examined, and each margin can be tested separately if necessary. However, the clinicopathologic details of the predictors of positive cytology result or lesions that developed recurrence at the surgical margin have not been fully examined.

In this study, we retrospectively analyzed data from patients undergoing ILC of autostapling cartridges in limited lung resection for pulmonary malignant tumors at our institution. Its efficacy in evaluating the cytologic status of the cut-end, predictors of positive cytology result, and subsequent recurrence at the surgical margin were also investigated.

## 2 Materials and methods

This study was approved by the Institutional Review Board (IRB) of the National Cancer Center, Japan (IRB No. 2017-105) in September 2017; the requirement for informed consent was waived. All extracted clinicopathologic data were obtained from our database. ILC was performed in 262 consecutive patients undergoing wedge or segmental resection for 311 lesions, including 139 primary lung cancers and 172 pulmonary metastatic tumors, between April 2004 and April 2010. We retrospectively reviewed the clinicopathologic features of patients with positive cytology results and those who developed recurrence at the surgical margin.

Surgical approach was thoracotomy in all cases. For pulmonary tumors metastasized from other primary sites, limited resection was the surgery of choice unless lobectomy was necessary to achieve complete resection. We usually secured a macroscopic resection margin >1 cm with the lung deflated [6, 17]. Limited resection for primary lung cancer was indicated as a compromise for high-risk patients and those with multiple lung cancers. The pulmonary function of these patients was often impaired by pulmonary fibrosis, which precluded lobectomy or stereotactic radiation therapy. For these patients, a tumor >2.0 cm was unavoidably resected if their macroscopic resection margin of >1 cm was secured. Patients with primary lung cancer for whom limited resection was intentionally indicated were participants in a prospective clinical trial targeting a tumor ≤2.0 cm depicted on high-resolution computed tomography (CT) as a ground -glass opacity (GGO) predominant nodule, which was radiologically suggestive of noninvasive or minimally invasive adenocarcinoma [6, 21, 22].

Disease stage was determined according to the 8th edition of the TNM classification [21]. Histological typing was determined according to the 4th edition of the World Health Association classification of tumors of the lung, pleura, thymus, and heart [22]. We evaluated vascular and pleural invasion using Victoria blue–van Gieson staining for all cases. Lymphatic permeation was usually evaluated with hematoxylin and eosin–stained slides. Tumor spread through alveolar spaces (STAS) was defined as the spread of lung cancer cells into air spaces in the lung parenchyma beyond the edge of a tumor [22], which was reported as an unfavorable prognostic factor [23].

Since 2004, we have performed ILC of autostapling cartridges to confirm negative surgical margin instead of frozen section analysis. All autostapling cartridges used for wedge or segmental resection of pulmonary malignancies are rinsed with 50 mL saline. The washing saline is centrifuged, and the sediment is stained using Papanicolaou's method to examine for cancer cells. The result is reported to the operating room within approximately 30 minutes, and in case of a positive result, additional wedge resection is usually attempted. If there is an anatomical restriction, additional segmental resection, lobectomy, or even pneumonectomy is performed if the patient is not at high risk.

Postoperative surveillance was performed using laboratory data and plain chest radiograph every 3–6 months as well as chest CT every 6–12 months. Patients were diagnosed with local recurrence when a tumor grew right on the cut-end staple line, and cell proliferation was confirmed by positron emission tomography–CT. Pathological confirmation was performed if possible but was not mandatory.

Fisher's exact test was used for contingency table analyses, and a p value of <0.05 was considered statistically significant. Analyses were performed using commercial software (JMP^®^ 12, SAS Institute Inc., Cary, NC, USA).

## 3 Results

Subjects consisted of 111 women and 151 men, with a median age at surgery of 66 years [interquartile range (IQR): 59–74 years]. The median size of the 311 lesions was 1.6 cm (IQR: 1.2–2.1 cm). We evaluated 139 primary lung cancers and 172 pulmonary metastatic tumors. Surgery for primary lung cancer was indicated in 85 patients as a compromise and in 54 patients as intentional resection. The reasons of compromised indication were the following: multiple tumors, 38; impaired pulmonary function, 27; and other high-risk condition, 20. Pulmonary metastatic tumors included 120 with a primary site from the colorectum, 10 from the lung, and 42 from other organs. Preoperative diagnoses of the 10 metastatic tumors from the lung were metachronous primary lung cancers based on their clinical courses, but the final pathological diagnoses were pulmonary metastases. Table 1 presents the characteristics of the 311 lesions.

**Table 1:** Lesion characteristics.

Characteristic	Primary lung cancer (147 lesions) (%)	Pulmonary metastatic tumor (170 lesions) (%)
Tumor size (cm)		
Median	1.7	1.5
IQR	0.6–4.0	0.4–3.7
GGO component		
Absent	72 (49)	170 (100)
Present	75 (51)	—
Clinical stage (UICC 8th)		
0	34 (23)	—
IA1	36 (25)	—
IA2	41 (28)	—
IA3	23 (16)	—
IB	11 (7)	—
>IIA	2 (1)	—
Surgical procedure		
Wedge resection	135 (92)	161 (95)
Segmentectomy	12 (8)	9 (5)
Primary site		
Colorectal	—	117 (69)
Head and neck	—	12 (7)
Lung	—	10 (6)
Others	—	31 (18)
Histological type		
Ad	117 (80)	137 (81)
Sq	20 (14)	14 (8)
Others	10 (6)	19 (11)
Autostapling cartridge lavage cytology		
Positive	11 (7)	11 (6)
Negative	136 (93)	159 (94)
Cut-end recurrence		
Present	8 (5)	3 (2)
Absent	139 (95)	167 (98)

IQR, interquartile range; GGO, ground -glass opacity; Ad, adenocarcinoma; Sq, squamous cell carcinoma.

**Fig. 1:**
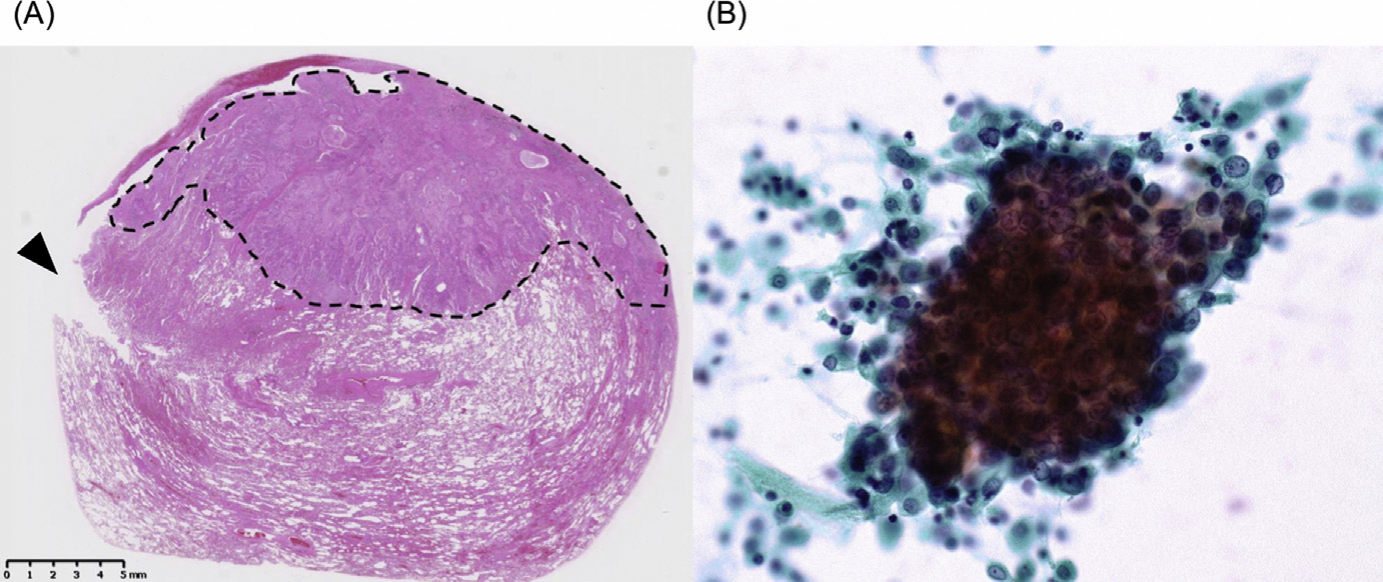
Representative example of a primary lung cancer with positive cytology result. This patient underwent wedge resection because of impaired pulmonary function. (A) Dotted lines indicate the solid nest of tumor cells and the arrowhead is the cut-end where stapled lung parenchyma was removed. The surgical margin is 0.2 cm. (Hematoxylin and eosin stain) (B) Intraoperative lavage cytology (ILC) of autostapling cartridges resulted in positive. (Papanicolaou stain).

Twenty-two (7%) lesions showed positive ILC results, including 11 primary lung cancers and 11 metastatic lung tumors. In postoperative pathological analysis, tumor cells were detected just on the margin where stapled lung parenchyma was removed in the 13 positive ILC lesions, and the resection margins of the remaining 9 lesions were negative despite a positive cytology result (Fig. 1). The median Microscopic margin length of these 9 lesions was 0.5 cm (IQR: 0.3–0.6 cm). None of the 289 negative ILC lesions had positive microscopic surgical margins in postoperative pathological analysis.

In primary lung cancers, tumor size (>2.0 cm, p < 0.001), pure solid tumor (p = 0.001), vascular invasion or lymphatic permeation (p < 0.001), visceral pleural invasion (p = 0.004), and STAS (p = 0.038) were significant predictors of a positive result (Table 2). In pulmonary metastatic tumors, tumor size (>2.0 cm, p = 0.016) and tumors originating from the lung (p < 0.001) were significant predictors of a positive result (Table 3).

**Table 2:** Predictors of positive lavage cytology result in primary lung cancer patients.

	No. of lesions		*P* value
	Lavage cytology negative (*n* = 136; %)	Lavage cytology positive (*n* = 11; %)	
Clinical tumor size			
≤2.0	97 (71)	1 (9)	<0.001
>2.0	39 (29)	10 (91)	
GGO component			
Absent	62 (46)	10 (91)	0.004
Present	74 (54)	1 (9)	
Surgical indication			
Intentional	53 (39)	3 (27)	0.53
Compromised	83 (61)	8 (73)	
Surgery			
Wedge resection	125 (92)	10 (91)	1
Segmentectomy	11 (8)	1 (9)	
Histologic type			
Ad	107 (79)	10 (91)	0.47
Sq	20 (15)	0 (0)	
Others	9 (6)	1 (9)	
Pathologic differentiation			
Well	74 (54)	1 (9)	0.004
Moderate/poor	62 (46)	10 (91)	
Lymphatic permeation or vessel invasion			
Positive	29 (21)	8 (73)	<0.001
Negative	107 (79)	3 (27)	
Pleural invasion			
Positive	14 (10)	5 (45)	0.006
Negative	122 (90)	6 (55)	
STAS			
Positive	43 (32)	7 (64)	0.045
Negative	93 (68)	4 (36)	

GGO, ground -glass opacity; Ad, adenocarcinoma; Sq, squamous cell carcinoma; STAS, tumor spread through alveolar spaces.

**Table 3:** Predictors of positive lavage cytology result in metastatic lung tumor patients.

Characteristic	No. of lesions		P value
	Lavage cytology negative (*n* = 159; %)	Lavage cytology positive (*n* = 11; %)	
Tumor size			
≤2.0	126 (79)	5 (45)	0.019
>2.0	33 (21)	6 (55)	
Surgery			
Wedge resection	151 (95)	10 (91)	0.46
Segmentectomy	8 (5)	1 (9)	
Primary site			
Colorectal	110 (69)	7 (64)	<0.001
Lung	6 (4)	4 (36)	
Others	43 (27)	0 (0)	
Histologic type			
Ad	126 (79)	10 (91)	0.7
Sq	14 (9)	1 (9)	
Others	19 (12)	0 (0)	
Lymphatic permeation or vessel invasion			
Positive	60 (38)	6 (55)	0.34
Negative	99 (62)	5 (45)	
Pleural invasion			
Positive	20 (13)	2 (18)	0.64
Negative	139 (87)	9 (82)	
STAS			
Positive	32 (20)	4 (36)	0.25
Negative	127 (80)	7 (64)	

Ad, adenocarcinoma; Sq, squamous cell carcinoma; STAS, tumor spread through alveolar spaces.

**Fig. 2:**
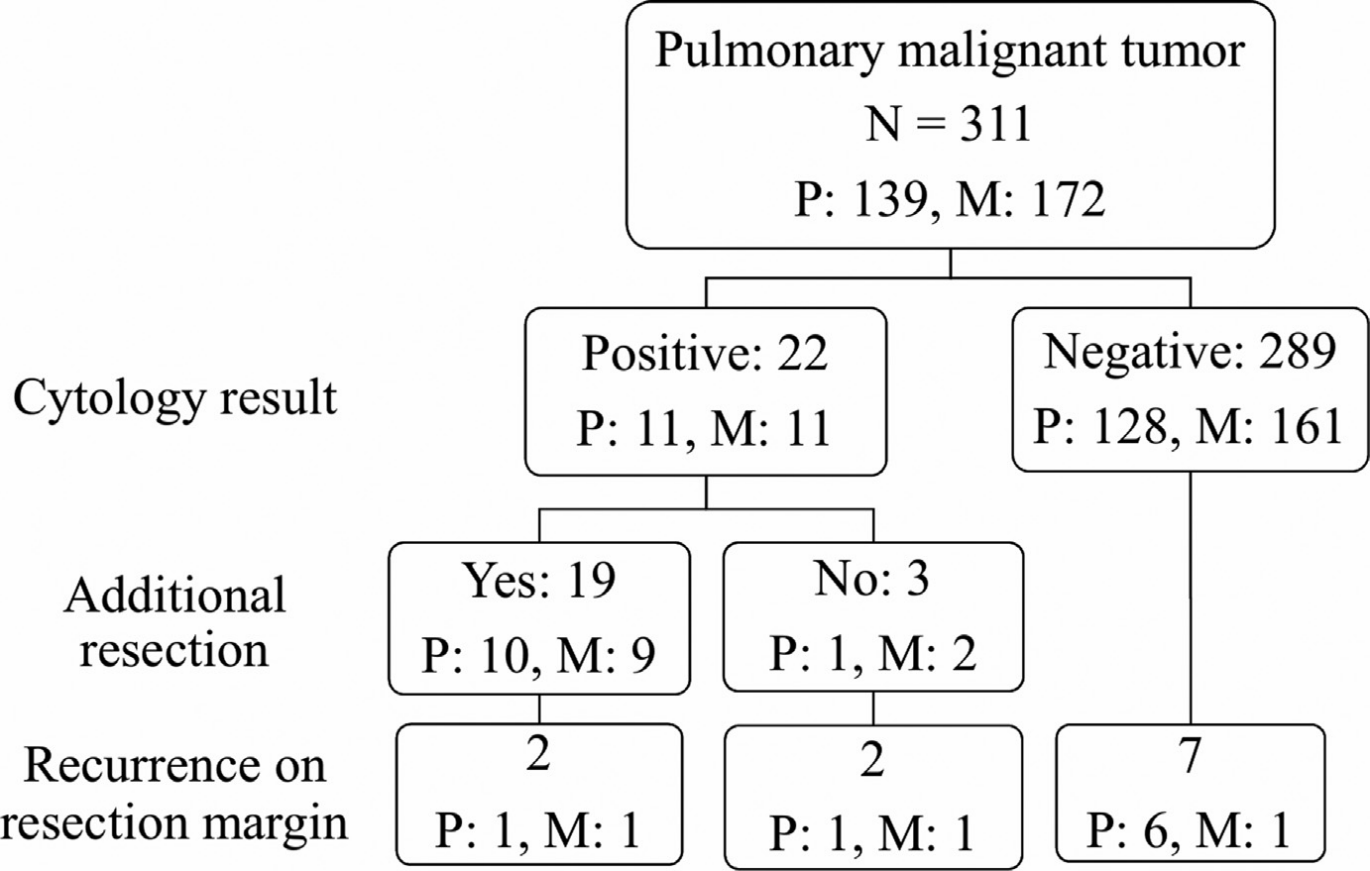
Surgical conversion and clinical course. P, primary lung cancer; M, pulmonary metastatic tumor.

Fig. 2 demonstrates the clinical course of all lesions in this study. Recurrence at the surgical margin developed in 11 (4%) lesions in total, and the recurrence rate was significantly higher in positive ILC lesions than in negative ones (18% vs. 2%, p < 0.001). The cut-ends of 19 of 22 positive ILC lesions were additionally resected. Of the 17 patients who initially underwent wedge resection, 11 underwent additional wedge resection, 1 segmentectomy, 4 lobectomy, and 1 pneumonectomy. Among 2 patients who initially underwent segmentectomy, 1 underwent additional wedge resection and 1 lobectomy. For the remaining 3 lesions, no additional resection was performed due to impaired respiratory function. Cytological evaluation was not repeated for most additionally resected specimens. In the patients who received additional anatomical resection of segmentectomy or greater, secured margin length was so substantial that cytological evaluation was not attempted. Because most patients who received additional wedge resection had impaired lung function, no further additional resection or cytological evaluation was indicated.

With the median follow-up period of 42 months (IQR: 30–65 months), recurrence at the surgical margin developed in 2 of the 19 lesions after additional resection (11%, 1 primary and 1 metastatic). Of the 3 lesions for which additional resection was abandoned, recurrence at the surgical margin developed in 2 (67%, 1 primary and 1 metastatic). Table 4 shows the detailed characteristics of the 4 patients who developed recurrence at the surgical margin. All 4 lesions were solid nodule resected by wedge resection and had any of vascular invasion, pleural invasion, or STAS. Among the 4 lesions, postoperative pathological analysis did not detect tumor cells on the resection margin in the 2 lesions, and in the additionally resected specimens in the 2 lesions, too. Cases 3 and 4 underwent salvage surgery for recurrence, and pathological findings similar to the initial lesions were confirmed. Among the 289 lesions showing a negative ILC result, recurrence at the surgical margin developed in 7 (2%, 6 primary and 1 metastatic). Table 5 shows the characteristics of these 7 lesions. The microscopic margin lengths were relatively short (median: 0.5 cm; IQR: 0.4–0.7 cm), and all 7 lesions showed no vascular invasion, pleural invasion, or STAS. The median time to recurrence was 48 months (IQR: 43–62 months). Cases 1, 4, 5, 6, and 7 underwent salvage surgery for recurrence, and pathological findings similar to the initial lesions were confirmed.

**Table 4:** Positive ILC patients developing cut-end recurrence.

Case	Primary or metastatic	Clinical tumor size (cm)	GGO component	Surgical margin (initial resection) (cm)	Additional resection	Tumor cells in the additional specimen	Histologic type	Vascular invasion Pleural invasion STAS	Time to cut-end recurrence
#1 Male, 75 y	Primary	3.6	Absent	1.0	No	—	Ad	V1, Ly0, PL1, STAS (–)	21 months
#2 Male, 75 y	Primary	2.4	Absent	0.1	Wedge resection	Absent	Ad	V0, Ly0, PL1, STAS (–)	16 months
#3 Male, 68 y	Metastatic (rectum)	1.9	Absent	0	No	—	Ad	V1, Ly0, PL0, STAS (–)	11 months
#4 Male, 70 y	Metastatic (rectum)	2.7	Absent	0	Wedge resection	Absent	Ad	V1, Ly1, PL1, STAS (+)	25 months

ILC, intraoperative lavage cytology; GGO, ground -glass opacity; Ad, adenocarcinoma; STAS, tumor spread through alveolar spaces.

**Table 5:** Negative ILC patients developing cut-end recurrence.

Gender, age	Primary or metastatic	Tumor size (cm)	GGO component	Surgical margin (cm)	Histologic type	Vascular invasion Pleural invasion Aerogenous spread	Time to cut-end recurrence
#1 Female, 70 y	Primary	0.8	Present	0.9	Ad	V0, Ly0, PL0, STAS (–)	48 months
#2 Male, 64 y	Primary	2.5	Present	1.3	Ad	V0, Ly0, PL0, STAS (–)	69 months
#3 Male, 71 y	Primary	2.8	Absent	0.3	Ad	V0, Ly0, PL0, STAS (–)	70 months
#4 Female, 74 y	Primary	1.6	Absent	0.4	Sq	V0, Ly0, PL0, STAS (–)	19 months
#5 Male, 64 y	Primary	2.5	Absent	0.4	Sq	V0, Ly0, PL0, STAS (–)	38 months
#6 Male, 66 y	Primary	1.5	Absent	0.5	Sq	V0, Ly0, PL0, STAS (–)	47 months
#7 Female, 71 y	Metastatic (lung)	1.4	Absent	0.5	Ad	V0, Ly0, PL0, STAS (–)	54 months

ILC, intraoperative lavage cytology; GGO, ground -glass opacity; Ad, adenocarcinoma; Sq, squamous cell carcinoma; STAS, tumor spread through alveolar spaces.

## 4 Discussion

In sublobar lung resection, sufficient surgical margin length in preventing local recurrence at the cut-end is essential [24], and we usually secure macroscopic resection margins >1 cm with the lung deflated [6, 17]. However, macroscopic safe surgical margins cannot always be obtained because of anatomical restriction. Even with the seemingly good surgical margin, we sometimes experience local recurrence after wedge or segmental lung resection.

The use of autostapling devices to cut and staple the lung parenchyma is widespread, and ILC of the autostapling cartridge used in limited lung resection is an effective technique to confirm a microscopically negative surgical margin during surgery [15, 16]. Higsahiyama et al. speculated that ILC of autostapling cartridges is superior to conventional techniques, such as stamped cytologic examination, because the entire area of the margins can be collectively examined and each margin can be separately tested if necessary with little contamination in a relatively short time [16].

To our knowledge, this is the first study to evaluate the relationship between detailed clinicopathologic features and the ILC result of autostapling cartridges based on a large number of lesions and long follow-up period. The positive ILC result rate of 7% was similar to that in previous reports [15, 16]. For patients with primary lung cancers, a tumor size of >2.0 cm and the absence of GGO were significant predictors of a positive ILC result. Researchers have reported the highly malignant nature of radiologically solid pulmonary nodules compared with GGO nodules [25, 26]. Avoiding sublobar resection for a radiologically solid nodule of large tumor size is recommended. The factors indicative of biological invasiveness (i.e., lymphatic permeation, vessel invasion, pleural invasion, and STAS) were also significant predictors of a positive ILC result. This is reasonable, considering the possible extension of a tumor cell spread in these more invasive tumors. When factors indicative of biological invasiveness are proven in final pathology, intensive follow-up is essential.

In patients with pulmonary metastatic tumors, a tumor size of >2.0 cm was a significant predictor of a positive ILC result. Obtaining wide surgical margins is essential in these patients, and ILC of autostapling cartridges is highly recommended during limited lung resection of a large lesion. It is unclear why factors indicative of biological invasiveness were not significant, unlike in patients with primary lung cancer.

Although various types of locoregional recurrence were included, the rates reported were >20% [4, 13] in patients with primary lung cancers and approximately 20% [12, 17] in patients with colorectal lung metastasis. In this study, the total incidence of recurrence at the surgical margin (4%) was lower than that previously reported in limited lung surgery, and the recurrence rate was significantly higher in positive ILC lesions than in negative ones. Careful surveillance is required especially after a positive cytology result, even though the surgical margin is negative in postoperative pathological analysis. The reduction in recurrence incidence by converting to more extensive resection in the case of positive cytology result was not proven to be statistically significant because of the small number of positive ILC cases. However, these data suggest that this technique is potentially useful in preventing recurrence at the resection margin.

In this series, 7 (2%) cases developed recurrence at the surgical margin despite a negative ILC result. None of these 7 lesions had pathological invasive findings predictive of positive ILC result. In 6 of the 7 lesions, the microscopic margin lengths were shorter than 1 cm. This may be the cause of remaining tumor cells at the cut-end, which were undetectable by ILC. The relatively longer time period to recurrence in the 7 lesions (median: 48 months; IQR: 43–62 months) compared with that of 4 local recurrence lesions with positive ILC result (median: 18.5 months; IQR: 15–22 months) might suggest that the lesions developed on the staple line are actually not local recurrence but metachronous multiple cancer. However, it is difficult to obtain hard differential diagnosis. For the lesions with microscopic margin length <1 cm, careful surveillance is recommended even after a negative ILC result, and long-term follow-up may also be required.

The limitation of this study is its retrospective nature. Although additional resections are clinically appropriate in case of positive ILC result, accurate interpretation of the influence of positive ILC on outcome becomes difficult. The pathologist was aware of the ILC result when examining the resected specimen. Although we confirmed that the microscopic surgical margins of the 289 ILC negative lesions were all negative, the microscopic margin lengths were not measured in all cases. We could not compare the margin lengths between the negative ILC lesions with recurrence and those without to evaluate if the short margin in those with recurrence was significant. The margin lengths are expected to be an important predictor of ILC result and recurrence at the surgical margin. However, the measured length does not always represent the shortest one because measurements were performed only on one microscopic slide for each tumor after removing stapled lung parenchyma. In this study, postoperative pathologic study did not detect any tumor cells solely on the margin in 9 of 22 positive ILC lesions and 2 of 4 lesions developing local recurrence after positive ILC. The ILC result may reflect the tumor cell status along the surgical margin more accurately than microscopic measurements of the margin lengths.

Patients with impaired pulmonary function may have less opportunity to detect occult recurrence events because they may not survive long enough to have a detectable recurrence. In this study, however, the median follow-up period of patients with compromised surgical indication was 38 months (IQR: 27–59 months), which was similar to that of fit patients. Even though the patients are so vulnerable that they are unfit for additional resection, we consider that ILC is useful because we expect that the early detection of local recurrence by intensive follow-up for the patients with positive ILC result increases the chance of salvage treatment.

## 5 Conclusions

ILC of autostapling cartridges in sublobar resection for primary or metastatic lung tumor was a powerful tool to evaluate the microscopic status of the entire surgical margin, and meticulous observation is required after a positive ILC result. In particular, intensive follow-up is essential for lesions as follows: pulmonary malignant tumors larger than 2.0 cm; primary lung cancers with radiologically solid appearance or pathologic factors indicative of biological invasiveness. A prospective study to elucidate the efficacy in preventing recurrence at the surgical margin is necessary.

## Declarations

### Author contribution statement

Tomohiro Miyoshi: Conceived and designed the experiments; Performed the experiments; Analyzed and interpreted the data; Contributed reagents, materials, analysis tools or data; Wrote the paper.

Junji Yoshida: Conceived and designed the experiments; Analyzed and interpreted the data; Contributed reagents, materials, analysis tools or data.

Keiju Aokage, Kenta Tane, Masahiro Tsubo: Contributed reagents, materials, analysis tools or data.

Genichiro Ishii: Performed the experiments; Analyzed and interpreted the data; Contributed reagents, materials, analysis tools or data.

### Funding statement

This research did not receive any specific grant from funding agencies in the public, commercial, or not-for-profit sectors.

### Competing interest statement

The authors declare no conflict of interest.

### Additional information

No additional information is available for this paper.
